# Chitosan-Modified Bentonite for the Adsorptive Removal of Three Organic Dyes: A Comprehensive Experimental and Theoretical Investigation

**DOI:** 10.3390/molecules31132224

**Published:** 2026-06-24

**Authors:** Teng Huang, Meng Li, Enwen Wang, Qian Wu

**Affiliations:** 1 School of Environment and Resource, Southwest University of Science and Technology, Mianyang 621010, China; 2 Key Laboratory of Waste Treatment and Resource Recycle, Southwest University of Science and Technology, Ministry of Education, Mianyang 621010, China; 3 School of Resources and Environmental Engineering, Anshun University, Anshun 561000, China

**Keywords:** chitosan modification, bentonite, adsorption performance, kinetic models, thermodynamic analysis

## Abstract

Chitosan has significant advantages due to its good biocompatibility and biodegradability. However, the original chitosan often has problems such as limited adsorption capacity and poor selectivity. Therefore, modifying chitosan to improve its adsorption performance has become an important research direction. This study adopted molecular dynamics simulations to explore the factors influencing the physical modification of chitosan and further conducted adsorption experiments with three different dyes. The results indicated that when combined with different clays, the interaction between chitosan and montmorillonite was more likely to occur, making it a suitable adsorbent for physical modification. As the molecular weight (MW) of chitosan increased, the <500 MW chitosan-modified bentonite exhibited the maximum adsorption capacity for different dyes, with the adsorption rates of 90.73% (methylene blue), 72.05% (methylene orange), and 32.28% (cresol red) at 20 mg/L, respectively. The adsorption kinetic and thermodynamic analysis indicated that the adsorption process is consistent with the pseudo-second-order kinetic model (R^2^ > 0.99004), while lower temperature was more conducive to the adsorption process. The results would help determine the optimum synthetic conditions for chitosan-based composite materials and make positive contributions to the green development of agriculture.

## 1. Introduction

Chitosan is a widely present biopolymer in nature, mainly derived from the shells of crustaceans such as shrimp and crabs, and often contains abundant functional groups such as hydroxyl (−OH) and amino (−NH_2_) groups [1,2]. Of particular note is that chitosan is non-toxic to crops, humans, livestock, and the environment, making it an ideal environmentally friendly adsorption material with extremely broad application prospects [3,4,5].

Chitosan is insoluble in water and most organic solvents. The original chitosan often has problems such as limited adsorption capacity and poor selectivity during the adsorption process. In order to promote the green development of agriculture and reduce environmental pollution, we urgently need to make more effective use of chitosan. Therefore, it is necessary to conduct in-depth research on the modification and application of chitosan. Clay, such as bentonite, kaolin, has the properties of high cation exchange capacity (CEC), plasticity and chemical stability [6,7,8]. Combining chitosan with various clays could improve the adsorption performance of the composite materials by increasing the specific surface area and internal porosity of the adsorbent [9,10,11,12]. Thus, chitosan/clay composite preparation is currently a widely used procedure for many kinds of applications [13,14,15,16,17]. However, current research may focus mostly on a specific modification method, with cumbersome experimental steps and limited improvement in performance. Research on the influencing factors, such as how the crystalline structure of minerals and the molecular weight of chitosan affect the adsorption properties, is still limited [18,19,20,21,22].

Compared to traditional experimental methods, theoretical calculations may possess the characteristics of wide applicability and a small workload, which is conducive to the development of the structural design and application of chitosan composite materials. In this work, we would investigate the factors influencing the physical modification of chitosan, such as different clay minerals and chitosan with different degrees of polymerization (DP), from the perspective of molecular dynamics simulation [23,24,25,26]. Combined with characterization and adsorption tests using the different dyes, including methylene blue, methylene orange and cresol red, the adsorption thermodynamics and kinetics were also investigated. The results could reveal the optimum modification conditions and the precise regulation of their adsorption properties with a more efficient approach, and help to provide strong support for the sustainable development of agriculture and the virtuous cycle of ecological environment [13,27,28,29].

## 2. Materials and Methods

### 2.1. Materials

Chitosan, which has a degree of deacetylation of 90%, was obtained from Shandong Mingsheng Biotechnology Development Co., Ltd. (Jinan, China). Bentonite (obtained from Guzhang Town Shanlinshiyu Mineral Products Co., Ltd., Guzhang County, China) had a montmorillonite (MMT) content greater than 90%. Glacial acetic acid (CH_3_COOH, MW = 60.05 g/mol, AR) was purchased from Chengdu Colon Chemical Co., Ltd. (Chengdu, China). Methylene blue (MB, MW = 373.90 g/mol, 98.5% in purity) was obtained from Tianjin Beichen Founder Reagent Factory (Tianjin, China). Methyl orange (MO, MW = 327.33g/mol, 96% purity) and cresol red (CR, MW = 382.43 g/mol, 96.8% purity) were purchased from Xilong Scientific Co., Ltd. (Guangzhou, China). Dialysis membranes (MD 34-500, MD 34-1000) were obtained from Henan Ruiruo Technology Co., Ltd. (Jiaozuo, China). All chemicals were of analytical grade and were used without further purification. Deionized distilled water was also used throughout the study.

### 2.2. Adsorption Experiments and Characterization

To validate the theoretical calculations regarding the modification effects of different DP on chitosan, dialysis membranes with varying MWs were selected for the adsorption experiments.

#### 2.2.1. Preparation of Chitosan with Different MWs

Two dialysis membranes with molecular weights of 500 and 1000 Da were selected, and the dialysis bags were boiled in ultrapure water for 30 min. Outside the dialysate was ultrapure water, while the inside was a mixture of 0.5 g chitosan and 50 mL of 5% (*v*/*v*) glacial acetic acid. Dialysis was performed under magnetic stirring at 500 rpm to obtain chitosan with different MWs. The dialysate was replaced and collected in the first 2 h, and then replaced and collected after 6 h and 12 h, respectively. The total duration of dialysis was 24 h [30,31]. Final products were the chitosan with the following molecular weights: <500, 500–1000 and >1000 Da.

#### 2.2.2. Preparation of Chitosan-Modified Bentonite

A total of 1 g of chitosan with different molecular weights was added to 1% glacial acetic acid solution, and 50 g of bentonite was stirred together with the solution in a constant-temperature water bath at 25 °C for 2 h. After drying at 105 °C to a constant weight, the chitosan-modified bentonite was obtained.

#### 2.2.3. Adsorption Experiments with Different Dyes

Different dyes were prepared and utilized in absorbance experiments. The characteristic UV-absorption peaks were observed at 664 nm for methylene blue, 464 nm for methyl orange, and 510 nm for cresol red [7,32]. Both unmodified and chitosan-modified bentonite samples were introduced into 20 mL of each dye solution. For the adsorption of different dyes, solutions of methylene blue, methyl orange, and cresol red (10 mg/L, 20 mg/L, 30 mg/L, 40 mg/L) were prepared, respectively. After moderate shaking (120 rpm) at room temperature for 24 h, the samples were centrifuged at 9000 rpm for 10 min (TG16G, Beihong Industrial Co., Ltd., Tai’an, China). The concentrations of the solutions before and after adsorption were determined using a UV–visible spectrometer (JH754PC, Jinghua Technology Instrument Co., Ltd., Shanghai, China). The adsorption capacity was determined as follows [33,34,35,36].

(1) $$\alpha =\frac{\left(C_{O}-C_{e}\right)\times 100\%}{C_{O}}$$

*α* is the adsorption rate (%), *Co* is the initial concentration (mg/L), and *Ce* is the concentration after adsorption (mg/L).

#### 2.2.4. FTIR Analysis

The functional groups were characterized via a Fourier transform infrared spectrometer (Thermo Nicolet 5700, Waltham, MA, USA) in the frequency range between 4000 and 400 cm^−1^ via the KBr pellet method.

### 2.3. Models and Calculation Method

#### 2.3.1. Calculation Method and Parameters

Based on our previous research [7,37,38], all calculations were performed using the DMol^3^ package in Material Studio 8.0 (MS) software. The equilibrium configuration at the lowest energy was determined through geometry optimization via the Becke–Perdew (BP) correction approximation under the generalized gradient approximation (GGA), with a smearing value set at 0.005 unless otherwise specified.

The initial unit cell was exported from the MS 8.0 software crystal structure database. The DMol^3^ module was used to optimize the crystal structure of different clays. We also identified the optimum crystal planes for different clay minerals [37,38,39]; therefore, kaolinite (001), MMT (020), and illite (020) were adopted for the following discussions to construct adsorption models with chitosan/chitin, separately.

The adsorption energy was calculated using the following equation:

*E_ads_ = E_total_* _(chitosan on clays)_ − (*E*_chitosan_ + *E_clays_*) (2)

*E^′^_ads_* = *E_total_* _(chitin on clays)_ − (*E*_chitin_
*+ E_clays_*) (3)

where *E_ads_* is the adsorption energy and *E_total (chitosan on clays)_* is the total energy of the different types of chitosan on the clays. For cases with less adsorption energy, adsorbability is more likely to occur [26,38,40,41].

#### 2.3.2. Calculation Models

**Figure 1:**
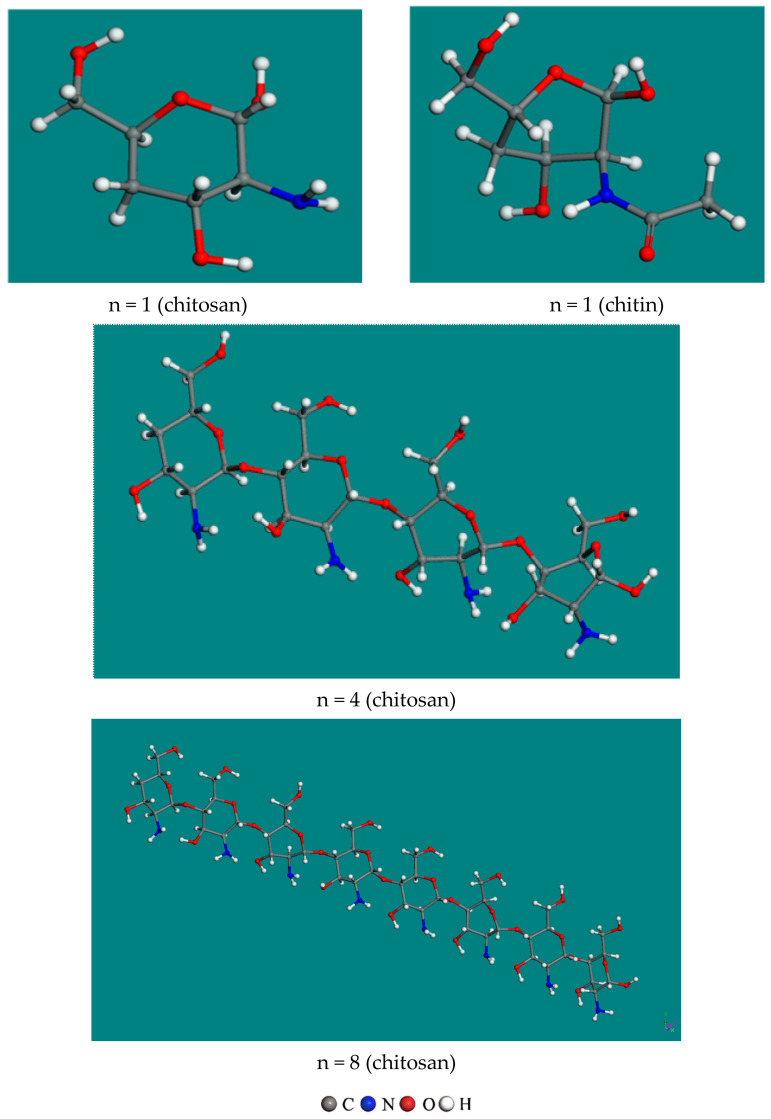
Chitosan and chitin with different degrees of polymerization after geometric optimization.

The models of chitosan/chitin with different degrees of polymerization after geometric optimization are listed in Figure 1.

## 3. Results and Discussion

### 3.1. Results of the Molecular Simulation

#### 3.1.1. Adsorption Energy of Clay Minerals and Chitosan/Chitin

**Figure 2:**
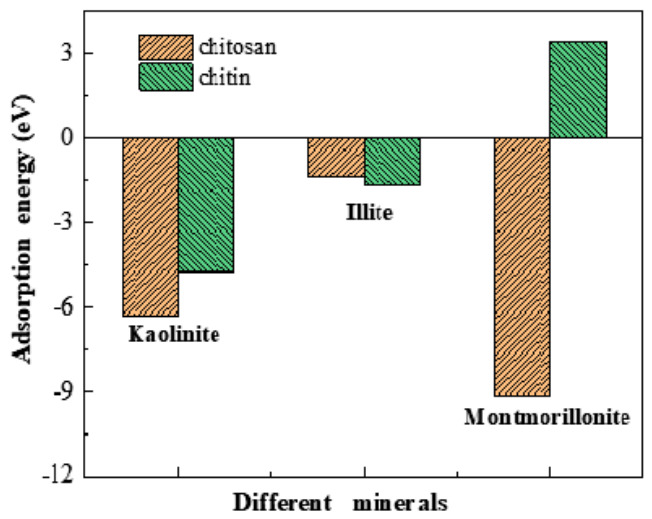
Comparison of the adsorption energies of different clays with chitosan/chitin.

The physical modification of chitosan can be achieved by combining clay minerals (such as kaolinite, montmorillonite, and illite) and other highly adsorptive substances. Therefore, this section will compare the adsorption energies between different clay minerals and chitosan/chitin, and also explore the adsorption order of different clays. The adsorption energies of different clay minerals with chitosan/chitosan were compared as shown in Figure 2.

To simplify the calculations, the DP of chitosan/chitin was set to one as discussed in this section. The adsorption energy of chitin with various minerals was generally higher than that of chitosan discussed in the text, indicating that the interaction between clay and chitosan is more favorable than with chitin. Among the adsorption energies with chitosan, MMT exhibited the lowest adsorption energy (−9.12 eV), which was significantly lower than kaolinite (−6.29 eV) and illite (−1.35 eV). Consequently, the interaction between chitosan and MMT is the most favorable, making MMT a promising candidate for physical modification. Therefore, subsequent investigations will focus on the adsorption characteristics of MMT in combination with chitosan.

#### 3.1.2. Adsorption Energy for Chitosan-Based Composites with Different DP

In this section, we would explore the adsorption properties of MMT with different DP of chitosan as listed in Table 1.

As MW of chitosan increased, the adsorption energy between MMT and chitosan would be raised. The adsorption energy (−9.12 eV) is the lowest when *n* = 1, while that may increase to −0.970 eV at *n* = 3. When *n* ≥ 4, the adsorption energy may be positive, indicating that chitosan modification might have a low efficiency at a relatively high MW.

**Figure 3:**
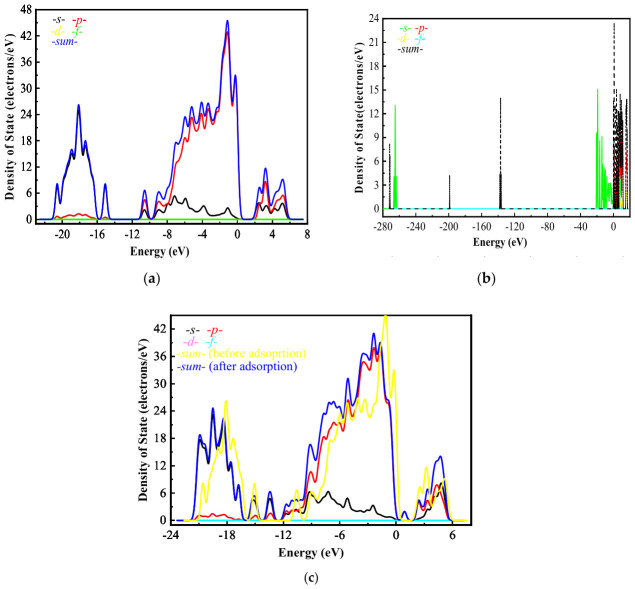
DOS analysis before and after adsorption between MMT and chitosan. (**a**) PDOS analysis of MMT before the adsorption; (**b**) PDOS analysis of chitosan before the adsorption; (**c**) PDOS and TDOS analysis after the adsorption between MMT and chitosan.

The density of states (DOS) could be considered as a visualization result for the band structure, representing the distribution of electron states in the energy space, and can also be characterized as the partial density of states (PDOS) and total density of states (TDOS). The DOS analysis before and after adsorption between MMT and chitosan (with *n* = 1) is presented in Figure 3.

Building upon our prior findings [37,38], we have calculated the PDOS to reveal the contribution of a specific atomic orbital for MMT as shown in Figure 3a. The DOS for chitosan (Figure 3b) was mainly concentrated in the range from −20 to 9 eV, which may display the majority of electron distribution. The peaks at about −375 eV and −265 eV were very sharp, indicating a much lower electron distribution. The sum of the bonding orbitals was attributed mainly to the *s* orbital at about −20 eV, −14~−11 eV, 6–7 eV, and the *p* orbitals at nearly −19 eV, −8~0 eV and 6–8 eV, whereas the *d* and *f* orbitals had little effect on the bond formation.

The TDOS would shift to a lower energy state after adsorption as presented in Figure 3c, whereas additional peaks appeared at nearly −20~−18 eV, −8~0 eV and 6 eV after adsorption, with intensities that were also greater than those before adsorption. The new peaks may be involved in charge transfer and orbital hybridization, indicating that there appears to be strong interaction between MMT and chitosan [37,39,40].

### 3.2. Adsorption Performance and Mechanism on Different Dyes

#### 3.2.1. Classification and FT-IR Analysis of Chitosan

To design in accordance with the different DP of chitosan as discussed in Section 3.1.2, dialysis membranes with MWs of 500 and 1000 were selected in this study, and the contents for the different MW ranges are presented in Table 2.

The component with a MW > 1000 had the highest content (61.62%), followed by the component with a MW ranging from 500 to 1000 (22.69%), while the MW less than 500 had the lowest content.

#### 3.2.2. Adsorption Performance for the Different MWs of Chitosan

**Figure 4:**
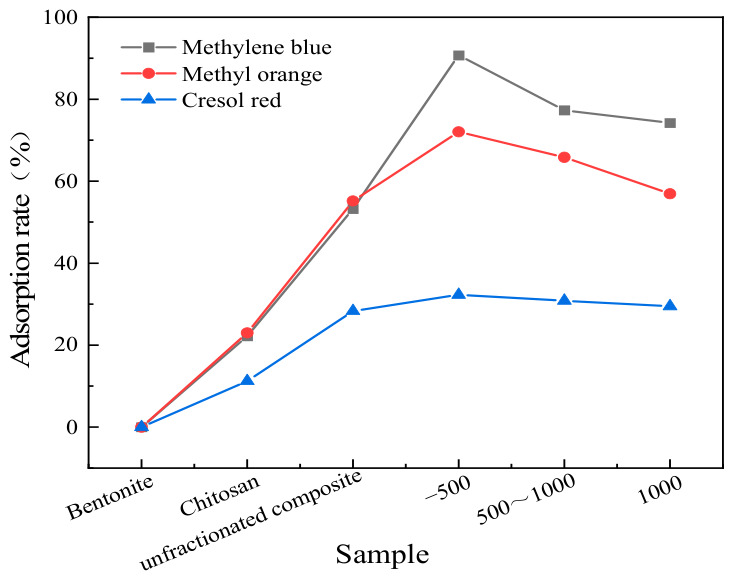
Adsorption performance of the three dyes at 20 mg/L.

Bentonite was modified with chitosan of different molecular weights as mentioned above. In this section, the adsorption experiments were carried out with an initial concentration of 20 mg/L for the different dyes, while the adsorption characteristics are presented in Figure 4.

There is virtually no adsorption towards the unmodified bentonite (raw ore). However, the classified chitosan may present a better adsorption performance. When the MW ≤ 500, chitosan-modified bentonite all exhibit the maximum adsorption efficiency, with the adsorption rates of 90.73% (methylene blue), 72.05% (methyl orange), and 32.28% (cresol red), respectively. By comparison, the 500–1000 MW group presented lower adsorption efficiencies of 77.32%, 65.81% and 30.84% for the different dyes. Meanwhile, the unfractionated chitosan-modified bentonite only achieved adsorption rates of 53.25%, 55.17% and 28.33%. The adsorption capacity may decrease as the molecular weight of chitosan increases [42,43,44], which further verify the theoretical calculation results shown in Section 3.1.2. Thus, MW could be considered as a good index to determine the modification effect of chitosan. The adsorption energy between MMT and chitosan would be raised as the molecular weight of chitosan increases, making the preparation of available chitosan-modified composites more difficult, while the adsorption performance of the composite for different dyes would be decreased accordingly [45,46,47].

**Figure 5:**
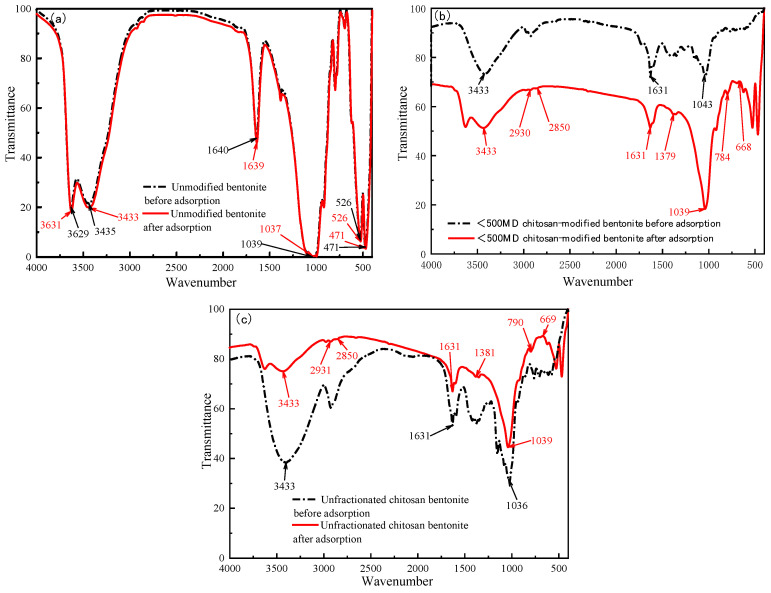
FT-IR analysis of modified-chitosan bentonite before and after adsorbing methylene blue. (**a**) unmodified bentonite. (**b**) MW < 500 chitosan-modified bentonite. (**c**) unfractionated chitosan-modified bentonite.

The FT-IR spectra of the various modified chitosan bentonite samples before and after adsorption were compared as shown in Figure 5. Obviously, no characteristic absorption peaks of methylene blue were observed in the spectra of bentonite before and after adsorption (Figure 5a), indicating that methylene blue may not be adsorbed onto the bentonite.

While the absorption peaks at 2930, 2931 cm^−1^ and 2850 cm^−1^ in Figure 5b,c may correspond to the −CH_2_− group, the absorption peak at 1379 and 1381 cm^−1^ is attributed to the stretching vibration of −CH_3_, and the absorption at 784 and 790 cm^−1^ corresponds to the external bending vibration of the aromatic benzene ring, indicating the characteristic absorption of methylene blue.

**Figure 6:**
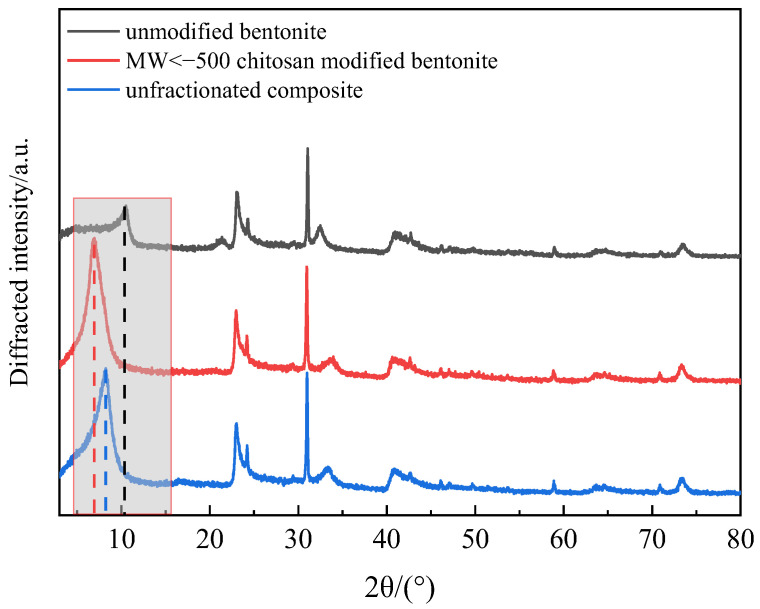
XRD analysis of bentonite before and after the modification.

XRD analysis of the raw ore and chitosan-modified bentonite is presented in Figure 6. The interlayer spacing of the sample was calculated using the following Bragg equation:

$$d_{\left(001\right)}=\frac{n}{2sin\theta }\lambda$$

*n* is the reflection order, which is 1; λ is the K_α_ radiation of Cu target, with a value of 1.5406 Å.

The *d*_(001)_ peak of chitosan-modified bentonite would shift to the lower angle. Combined with the calculation of interlayer spacing as presented in Table 3, the chitosan molecules may enter the interlayer of bentonite during the modification process. Further analysis revealed that the MW < 500 chitosan-modified bentonite may possess a larger interlayer spacing (12.69 Å), indicating that chitosan with low molecular weight is more easily combined with the bentonite.

**Figure 7:**
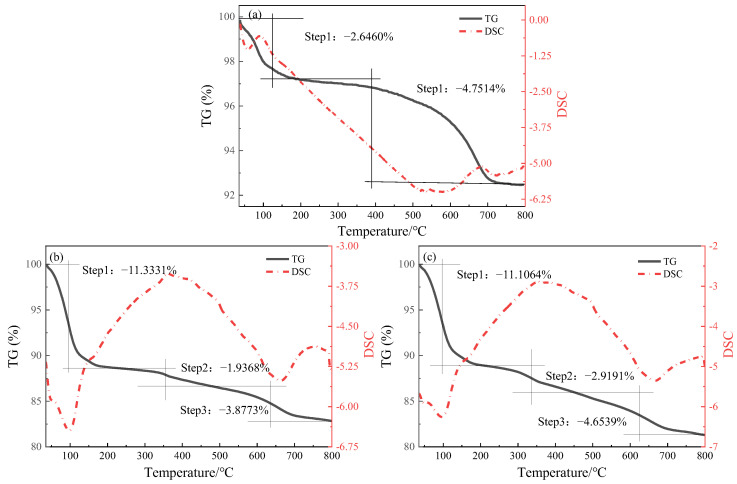
TG-DSC analysis of bentonite before and after modification. (**a**) Unmodified bentonite; (**b**) chitosan-modified bentonite before adsorption; (**c**) chitosan-modified bentonite after adsorption.

The thermal properties of bentonite before and after modification was conducted with a TG-DSC analyzer as presented in Figure 7. The weight loss of unmodified bentonite mainly consists of two stages. Due to the evaporation of adsorbed water and a small amount of free water in the bentonite, the mass slightly decreases from room temperature to 150 °C. The weight loss changes obviously after 450 °C, corresponding to the removal of structural water from bentonite. While the weight loss of chitosan-modified bentonite could primarily divide into three stages. The weight loss may significantly increase compared to the unmodified bentonite (Figure 7a) from room temperature to 150 °C, primarily due to the evaporation of adsorbed water and the initial dehydration decomposition of chitosan loaded on the surface of bentonite. The weight loss from 200 °C to 450 °C is mainly attributed to the thermal decomposition of chitosan, indicating that the chitosan molecules have penetrated the interlayer of bentonite, forming a stable composite structure. Further analysis revealed that the weight loss of modified bentonite composite was more remarkable after dye adsorption (methylene blue), demonstrating that the dye molecules thermally decomposed along with chitosan at high temperatures, leading to the cleavage of functional groups and degradation of molecular chains. The weight loss in the range of 450 °C to 800 °C was almost equal to the unmodified bentonite, which may confirm that the adsorption process would not affect the crystal structure of bentonite, ensuring the excellent structural stability of the prepared composite.

**Figure 8:**
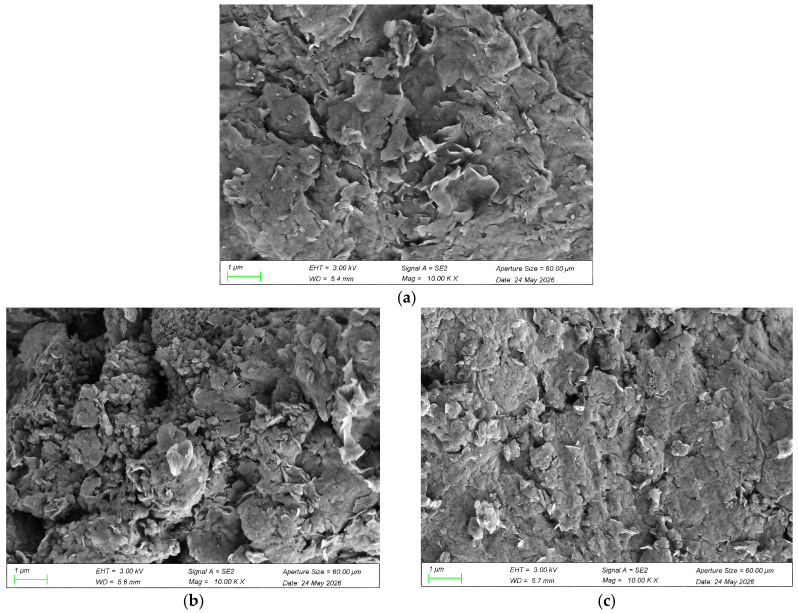
SEM analysis of bentonite before and after modification. (**a**) Unmodified bentonite; (**b**) chitosan-modified bentonite before adsorption; (**c**) chitosan-modified bentonite after adsorption.

SEM analysis of different samples before and after adsorption is presented in Figure 8. Raw ore of bentonite exhibits a typical layered structure, with curled and loose edges, as well as numerous gaps and pores between the layers. The amorphous chitosan is coated on the surface of bentonite layers and fills part of the interlayer gaps (Figure 8b). Mineral flakes are agglomerated by polymer bonding, and many organic mesoporous are constructed. The surface roughness and internal pores of the composite are filled with the dye molecules after adsorption, and the surface tends to be dense and flat, which may confirm that chitosan modification can optimize the pore structure and introduce the organic active sites, effectively capturing the methylene blue through pore retention and functional group binding.

#### 3.2.3. Adsorption Capacity Under Different Temperatures and Thermodynamic Analysis

**Figure 9:**
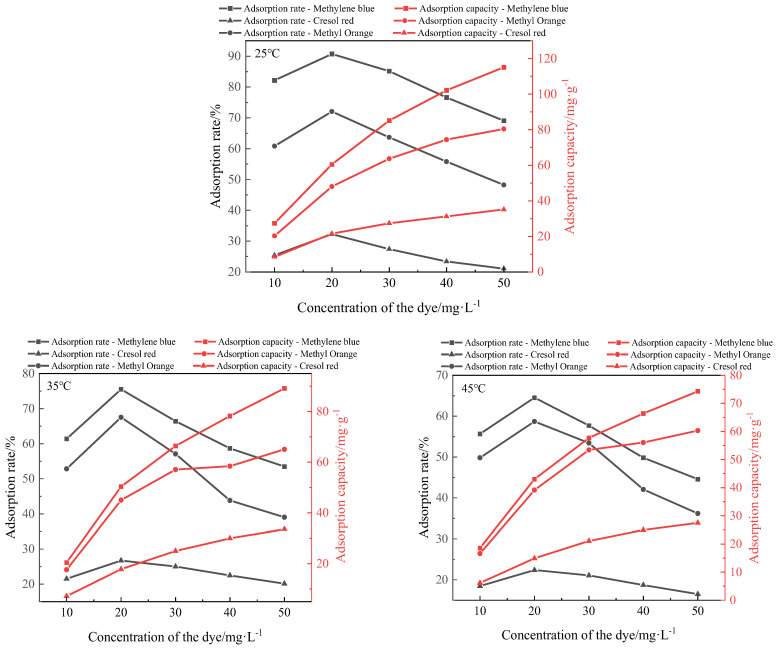
Adsorption rates and capacity by modified bentonite under different temperatures.

**Figure 10:**
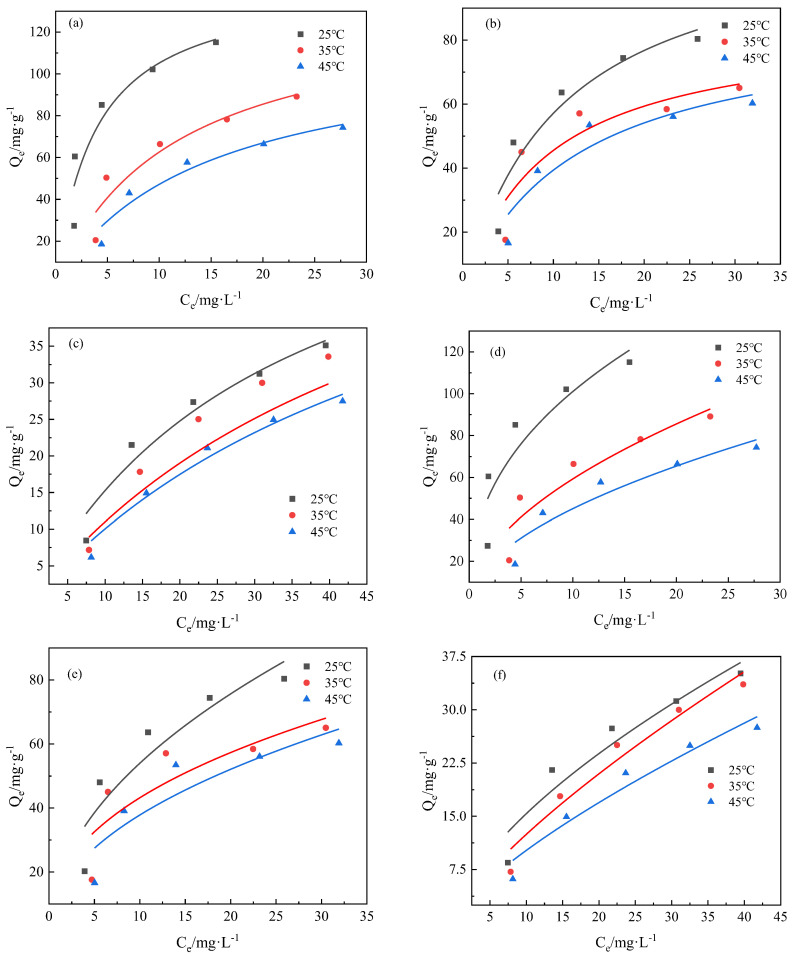
Adsorption isothermal models of modified bentonite for three dyes. (**a**) Methylene blue-Langmuir. (**b**) Methyl orange-Langmuir. (**c**) Cresol red-Langmuir. (**d**) Methylene blue-Freundlich. (**e**) Methyl orange-Freundlich. (**f**) Cresol red-Freundlich.

As described in Section 2.2.3, 0.3 g of chitosan-modified bentonite with a molecular weight of 500 Da was added to the different dye solutions with mass concentrations of 10 mg/L, 20 mg/L, 30 mg/L, 40 mg/L, and 50 mg/L, respectively. The adsorption rates and adsorption capacity under different temperatures are illustrated in Figure 9 and Figure 10. The experimental data were fitted using the Langmuir (4) and Freundlich (5) adsorption isotherm models, respectively, while the results for the different dyes are shown in Table 4.

(4) $$q_{e}=\frac{q_{m}K_{L}C_{e}}{1+K_{L}C_{e}}$$

(5) $$q_{e}=K_{F}C_{e}$$

In the formula, *C_e_* represents the mass concentration of dyes at the adsorption equilibrium, measured in mg/L; *q_m_* represents the maximum adsorption capacity, measured in mg/g; *K_L_* is the Langmuir adsorption equilibrium constant; *K_F_* is the Freundlich adsorption equilibrium constant.

The maximum adsorption amounts of methylene blue, methyl orange, and cresol red are 115.13 mg/g, 80.42 mg/g, and 35.12 mg/g, respectively. Methylene blue may permanently maintain the highest adsorption rate with changes in concentration and temperature, followed by methyl orange. For the concentration of 20 mg/L, the adsorption rate of methylene blue may decrease from 90.73% to 64.52%, and the adsorption rate of methyl orange may decrease from 72.05% to 58.69% when the temperature rises. This demonstrates that low temperature may be favorable to adsorption, while the adsorption rates may also decrease gradually with the increase in dye concentrations.

*R*^2^ values of the Langmuir adsorption isotherm model for methylene blue, methyl orange, and cresol red are all greater than those of the Freundlich adsorption isotherm model. This may indicate that the adsorption process belongs to uniform adsorption on a single molecular layer, and the dyes are orderly arranged between the layers of the modified bentonite. The *K_F_* values may decrease with the increase in temperatures, indicating that the increase in temperature may lead to a decrease in the adsorption rate and is not conducive to the adsorption process.

Thermodynamic analysis could also further cognize the adsorption mechanism for the different dyes, while the thermodynamic parameters at different temperatures could be calculated using the following Formulas (6)~(8).

(6) $$∆G=-RT\ln\frac{q_{e}}{C_{e}}$$

(7) ∆G=∆H−T∆S

(8) $$\ln\frac{q_{e}}{C_{e}}=\frac{∆S}{R}-\frac{∆H}{R}\times \frac{1}{T}$$

***∆G*** is Gibbs free energy, kJ/mol; ***∆H*** is enthalpy change, kJ/mol; ***∆S*** is entropy change, kJ/(mol·K); ***R*** is the ideal gas constant, 8.314 J/(mol·K); and ***T*** is temperature in K.

**Figure 11:**
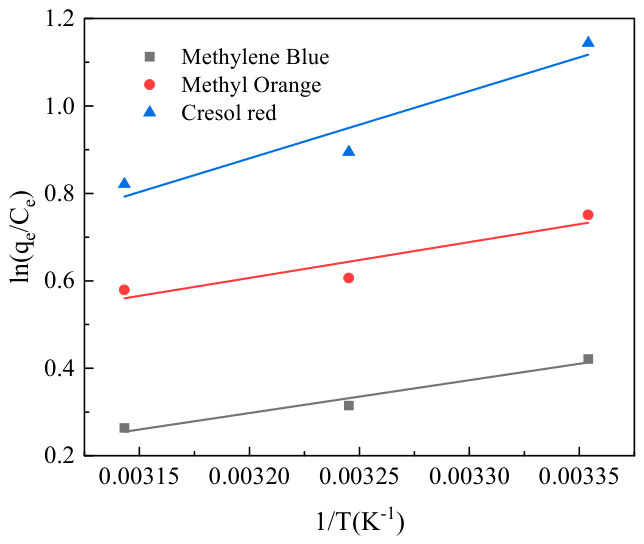
Adsorption thermodynamics analysis for the different dyes.

The thermodynamic analysis and calculated parameters for the different dyes are presented in Figure 11 and Table 5. ∆G of the samples at different temperatures are negative, indicating that the adsorption process is spontaneous. The absolute value of ∆G would decrease with the rise in temperature, indicating that the adsorption is retarded, and both the adsorption capacity and adsorption rate would decrease with the increase in temperature. The ∆H value < 0 indicates that the adsorption is an exothermic process, and lower temperature will cause the modified bentonite to adsorb more dyes [20,40,48,49].

#### 3.2.4. Adsorption Capacity Under Different pH Values

**Figure 12:**
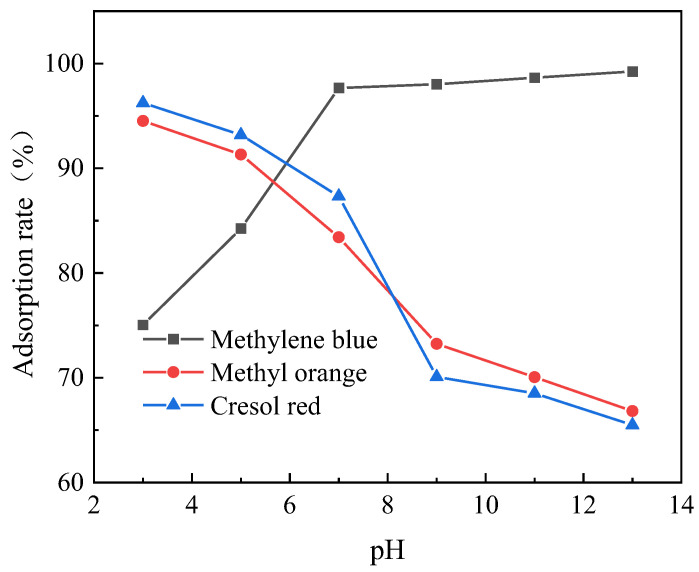
Adsorption rates of the modified bentonite under different pH values.

A total of 0.3 g of chitosan-modified bentonite with a molecular weight of 500 Da was added to the different dye solutions, with a mass concentration of 20 mg/L under different pH conditions (pH = 3, 5, 7, 9,11, 13), while the adsorption rates under different pH values are presented in Figure 12.

The adsorption rate for methylene blue rises with the increase in pH values as the surface of the modified bentonite is positively charged under acidic conditions, which has electrostatic repulsion with the cationic groups of methylene blue. As the pH value of the solution gradually increases, the electrostatic interaction decreases, thus the adsorption rate could be increased for the methylene blue. However, the adsorption rates for methyl orange and cresol red solutions would decrease with the increase in pH values. As methyl orange and cresol red have anionic functional groups, which easily adsorbed by the modified bentonite under acidic conditions, resulting in higher adsorption rates, the negatively charged functional groups of the modified bentonite will have electrostatic repulsion under alkaline conditions with the above dyes, thus reducing the adsorption rate for methyl orange and cresol red.

#### 3.2.5. Adsorption Time and the Kinetics Analysis

**Figure 13:**
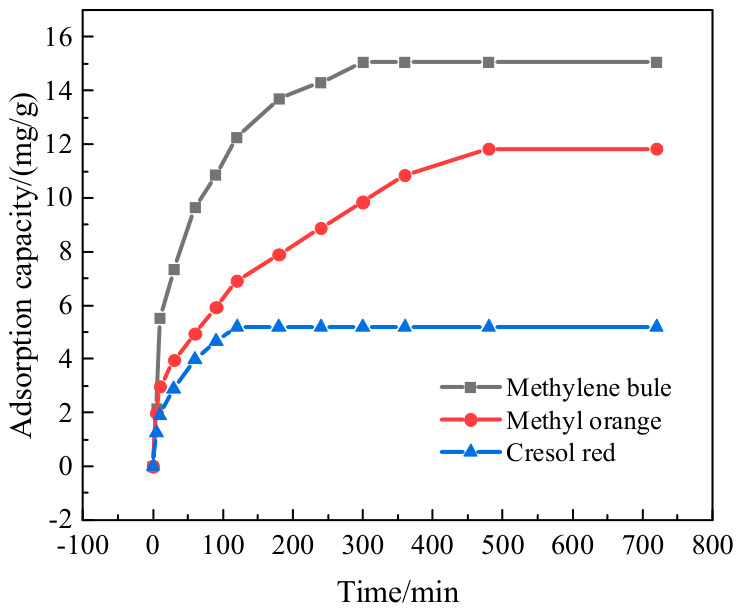
Relationship between the adsorption amount and adsorption time for the different dyes.

Figure 13 presented the adsorption with the MW < 500 chitosan-modified bentonite over different adsorption times. The adsorption amount increases with the extension of adsorption time for different dyes, and the highest adsorption amount could be 15.06 mg/g for the methylene blue; for methyl orange and cresol red, they were 11.82 mg/g and 5.18 mg/g, respectively. The final adsorption equilibrium times for the methylene blue, methyl orange, and cresol red could be 300, 480, and 120 min, respectively.

To determine adsorption kinetic characteristics for the different dyes, the obtained data were fitted using the following pseudo-first-order kinetic Equation (9) and pseudo-second-order kinetic Equation (10).

(9) $$\ln\left(q_{e}-q_{t}\right)=\ln q_{e}-k_{1}t$$

(10) $$\frac{t}{q_{t}}=\frac{1}{k_{2}q_{e}^{2}}+\frac{1}{q_{e}}t$$

***q_e_*** and ***q_t_*** are the adsorption capacities at equilibrium time and time t, respectively, mg/g; ***t*** is the adsorption time, min; ***k*_1_** is the pseudo-first-order kinetic rate constant, min^−1^; and ***k*_2_** is the pseudo-second-order kinetic rate constant, g/(mg·min).

**Figure 14:**
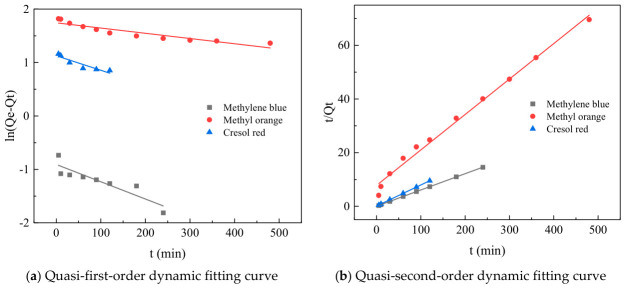
The kinetics and fitting curves of the three different dyes.

The adsorption kinetic parameter and fitting curves are presented in Figure 14 and Table 6. The final adsorption equilibrium times of the modified bentonite for methylene blue, methyl orange, and cresol red are 240, 480, and 120 min, respectively. The adsorption process for the three dyes fits poorly with the pseudo-first-order kinetic model (R^2^ < 0.84909) and is more consistent with the pseudo-second-order kinetic model (R^2^ > 0.99004). This indicates that the reduction in dye concentration in the solution is due to interlayer ion exchange, and the adsorption process is mainly chemical adsorption [7,49,50,51].

**Figure 15:**
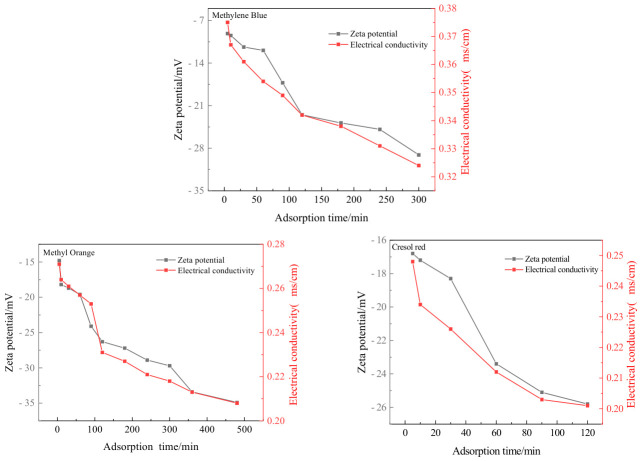
Zeta potential and electrical conductivity for the different adsorption times.

The change in Zeta potential reflects flocculation stability, rheological property of the colloidal dispersion system and the interaction relationship of the solution system. A higher absolute value of Zeta potential indicates homogeneous repulsion between colloidal particles and good dispersion state, and a lower absolute value of Zeta potential may determine that colloidal particles are easy to agglomerate and flocculate [7,27,52]; the Zeta potential value and conductivity at different adsorption time are presented in Figure 15.

With the increase in adsorption time, both the Zeta potential value and conductivity decrease, while the absolute value of the Zeta potential may increase, indicating that the dispersion state of the solution is improved. The changes in the absolute value in methylene blue are more significant, which could be more advantageous for improving adsorption performance.

#### 3.2.6. Infrared Spectra Analysis Before and After Adsorption

**Figure 16:**
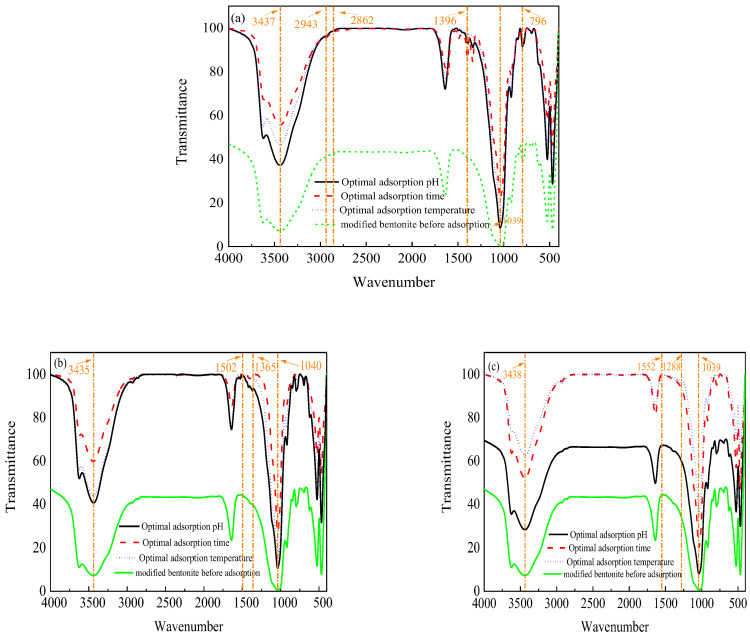
Infrared spectra of modified bentonite before and after adsorption. (**a**) Methylene blue. (**b**) Methyl orange. (**c**) Cresol red.

Figure 16 presented the comparison of infrared spectra under the optimal adsorption conditions as discussed above. Comparing to the bentonite before adsorption, the absorption peaks at 2943 cm^−1^ and 2862 cm^−1^ correspond to the −CH_2_− group in Figure 16a, while the absorption peak at 1396 cm^−1^ and 796 cm^−1^ may correspond to the stretching vibration peak of −CH_3_, and out-of-plane bending vibration peak of =C−H on the aromatic benzene ring, which could strongly prove that the modified bentonite has adsorbed methylene blue. The peak at 1502 cm^−1^ is the stretching vibration peak of the azo group (-N=N-) in Figure 16b, and the absorption intensity at 1365 cm^−1^ may belong to the stretching vibration peak of sulfonic acid group (-SO_3_^−^), which also verifies that the modified bentonite has adsorbed methyl orange.

### 3.3. Performance of the Adsorption/Desorption Cycles

**Figure 17:**
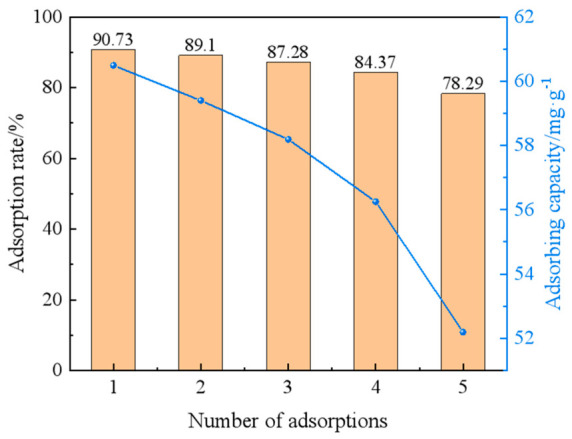
Cyclic performance of the adsorbent for methylene blue.

To further investigate the regeneration stability of the chitosan-modified bentonite adsorbent, five consecutive adsorption/desorption cycles experiments were conducted according to Section 2.2.3. 0.3 g adsorbent was added, with the initial concentration of methylene blue of 20 mg/L and temperature 25 °C. Subsequently, 0.1 mol/L NaOH solution was used for desorption after the adsorption saturation, followed by centrifugation, water washing and drying before entering the next round of adsorption. The cyclic performance is presented in Figure 17.

Adsorption rate for the first adsorption can reach 90.73%, with an adsorption capacity of 60.49 mg/g. As the number of cycles increases, the adsorption performance shows a slow decline trend, with the adsorption rates of 89.10%, 87.28%, and 84.37% for the second, third, and fourth cycles, respectively. The adsorption capacity remained at 52.19 mg/g after five complete regeneration cycles, which is a decrease of only about 13.7% compared to the first time.

## 4. Conclusions

By fully utilizing the abundant natural resource, chitosan is expected to play a greater role in future agricultural production. This study investigated the factors influencing the physical modification of chitosan from the perspective of molecular dynamics simulations, combing with adsorption tests and characterization using different dyes. The primary conclusions are as follows:

(1) Among the adsorption energies with chitosan, MMT exhibits the lowest value (−9.12 eV). This indicates that, when combined with various clays, the modification reaction between chitosan and MMT is more likely to occur, making it a suitable adsorbent for physical modification.

(2) As the MW of chitosan increased, the adsorption energy between MMT and chitosan would be raised. The adsorption performance was also verified using different dyes. The MW < 500 chitosan-modified bentonite all exhibited the maximum adsorption capacity for the different dyes; the maximum adsorption amounts of methylene blue, methyl orange, and cresol red are 115.13 mg/g, 80.42 mg/g, and 35.12 mg/g, respectively.

The adsorption rates may also decrease gradually with the increase in dye concentrations, with adsorption rates of 90.73% (methylene blue), 72.05% (methylene orange), and 32.28% (cresol red) at 20 mg/L. Thus, MW could be a good index for determining the order of adsorption for the modification effect of chitosan.

(3) The adsorption kinetic analysis indicated that the adsorption process is more consistent with the pseudo-second-order kinetic model (R^2^ > 0.99004). Thermodynamic analysis suggested that the adsorption process is spontaneous, while lower temperature was more conducive to the adsorption process. Results could help to develop and synthesize the chitosan-modified composites, which can achieve the precise regulation for the adsorption performance, also further promoting the application of chitosan in sustainable agricultural development and ecological environment. Adsorption/desorption cycles experiments indicated that the adsorption capacity remained at 52.19 mg/g after five complete regeneration cycles, with a decrease of only about 13.7% compared to the first time.

## Figures and Tables

**Table 1:** Adsorption energy between MMT and chitosan with different DP.

	Degree of Polymerization (DP)	Molecular Formula	Adsorption Energy (eV)
(a)	*n* = 3	(C_6_H_11_NO_4_)_3_, MW ≈ 483	−0.970
(b)	*n* = 4	(C_6_H_11_NO_4_)_4_, MW ≈ 644	>0
(c)	*n* = 6	(C_6_H_11_NO_4_)_6_, MW ≈ 966	>0

**Table 2:** The contents for different molecular weight ranges.

Molecular weight (MW)	<500	500–1000	>1000
Content (%)	15.69	22.69	61.62

**Table 3:** The interlayer spacing of bentonite before and after the modification.

Sample	2θ/°	*d*_(001)_/Å
Unmodified bentonite	10.52	8.40
MW < 500 chitosan-modified bentonite	6.96	12.69
Unfractionated composite	8.06	10.96

**Table 4:** Parameters of the adsorption isothermal model.

Different Dyes	Temperature/°C	Langmuir			Freundlich		
		*q_m_*/(mg·g^−1^)	*K_L_*	*R* ^2^	1/*n*	*K_F_*	*R* ^2^
Methylene blue	25	145.1739	0.2632	0.8820	0.4106	39.2567	0.8346
	35	134.7296	0.0868	0.8971	0.5299	17.4904	0.8640
	45	115.3488	0.0692	0.9409	0.5383	13.0446	0.8980
Methyl orange	25	116.9191	0.0956	0.9084	0.4867	17.6095	0.8531
	35	85.2195	0.1148	0.8044	0.4082	16.8793	0.7343
	45	86.5505	0.0835	0.8825	0.4622	13.0493	0.8074
Cresol red	25	65.7161	0.0303	0.9508	0.6330	3.5782	0.9208
	35	70.3054	0.0187	0.9729	0.7526	2.2016	0.9555
	45	67.0379	0.0176	0.9716	0.7320	1.8897	0.9514

**Table 5:** Thermodynamic parameters of the adsorption.

Samples	T/K	*ΔH/*KJ·mol^−1^	*ΔS/*kJ·(mol·K)^−1^	*ΔG/*KJ·mol^−1^
Methylene blue	293.15			−1.0448
	303.15	−6.2489	−0.0175	−0.8069
	313.15			−0.6971
Methyl orange	293.15			−1.8614
	303.15	−6.8166	−0.0168	−1.5539
	313.15			−1.5322
Cresol red	293.15			−2.8358
	303.15	−12.7977	−0.0336	−2.2917
	313.15			−2.1723

**Table 6:** Adsorption kinetic parameters.

Parameter of Dynamic Model	Different Dyes		
	Methylene Blue	Methyl Orange	Cresol Red
pseudo-first-order kinetic			
*q_e_*/mg·g^−1^	0.40337	5.7166	3.08680
*K*_1_	0.00323	0.00098	0.00272
*R* ^2^	0.78566	0.84909	0.81637
pseudo-second-order kinetic			
*q_e_*/mg·g^−1^	16.47718	7.56659	12.71941
*K*_2_	0.16942	0.00224	0.06307
*R* ^2^	0.99997	0.99004	0.99991

## Data Availability

The original contributions presented in this study are included in the article. Further inquiries can be directed to the corresponding author.
